# Poor radiological outcomes and associated factors among tibial shaft fracture patients treated with intramedullary nail fixation at Addis Ababa Burn, Emergency and Trauma Hospital, Ethiopia

**DOI:** 10.3389/fsurg.2025.1473038

**Published:** 2025-02-04

**Authors:** Yohannes Shugie, Samuel Kebede, Fanna Adugna, Dereje Bayissa Demissie, Tilahun Desta

**Affiliations:** Department of Orthopedics Surgery, St. Paul’s Hospital Millennium Medical College, Addis Ababa, Ethiopia

**Keywords:** tibial fracture, SIGN nail, non-union, union, intramedullary, fixation, RUST score

## Abstract

**Background:**

Tibial shaft fractures account for the majority of operatively treated long bone fractures and have the greatest prevalence of open wounds. For both open and closed injuries, intramedullary fixation has become the standard of therapy. At Addis Ababa Burn, Emergency, and Trauma (AaBET) Hospital, the rates of poor radiological outcomes for tibial shaft fractures treated with intramedullary fixation are unknown.

**Methods:**

A retrospective health facility-based cross-sectional study design was conducted among patients with tibial shaft fractures treated with intramedullary nails at AaBET Hospital. Data were collected by reviewing medical records and x-rays. The study was conducted on a sample size of 160 using a simple random sampling technique. Descriptive statistics such as frequency and percentage were used to summarize the results. Binary logistic regression was used to describe the associations between variables. A *P*-value < 0.05 was considered statistically significant.

**Results:**

This study included 122 (76.3%) men and 38 (23.8%) women with a mean age of 36.3 ± 13.9. The magnitude of poor radiological outcomes among the tibial shaft fracture patients treated with intramedullary nail fixation was 23.1%, with factors including include the presence of medical comorbidity [adjusted odd ratio (AOR) (95% confidence interval, CI): 16.5 (2.524–108.69)], having diabetes mellitus [AOR (95% CI): 3.85 (1.07–14.08)], Gustilo–Anderson type III (GA III) open fractures [AOR (95% CI): 17.4 (3.11–97.72)], and post-operative infection [AOR (95% CI): 13.9 (5.8–33.16)] identified as being significantly associated with poor radiological outcomes.

**Conclusion:**

The magnitude of poor radiological outcomes in this study is comparable to other similar studies. The study found that factors including Gustilo–Anderson type III open fractures, diabetes mellitus, and post-operative infections increase the odds of poor radiological outcomes in patients with tibial shaft fractures after intramedullary nailing. Therefore, surgeons should improve their assessment and evaluation of patients with infection signs and use negative wound pressure for GA III fractures.

## Background

The tibial diaphysis is triangular in cross-section with very thick cortices and is extremely strong. Cortical thickness tends to decrease with age, predisposing the tibia to lower energy injury mechanisms. The tibia is the main weight-bearing bone in the leg, carrying greater than 80% of the load (1).

Tibial shaft fractures are most commonly seen in road traffic accidents and are associated with a wide range of injury mechanisms and severities. Tibial shaft fractures are the most common long bone fractures, with roughly 63% of tibia fractures being open fractures, and management is greatly influenced by the associated soft tissue injury. Because one-third of the tibia's surface is subcutaneous, open fractures are more likely than in any other long bone. Delayed union, malunion, non-union, and infections are common complications of tibial shaft fractures (2, 3).

The goal of tibial shaft fracture treatment is to achieve early autonomous ambulation by obtaining union with proper alignment and restoration of normal knee and ankle mechanics. This is performed by reamed or unreamed intramedullary nailing, open reduction and internal fixation (ORIF), and external fixation, either non-operatively or surgically. Currently, however, intramedullary devices have become the standard option for both open and closed injuries (3).

The treatment of open tibial fractures is difficult and often controversial with no general consensus on their management. The complication rate rises exponentially with high-energy trauma, soft tissue disruption, wound contamination, altered vascularity, and unstable fractures. Several strategies have been developed to minimize these complications and include the use of prophylactic antibiotics, tetanus toxoid, immediate soft tissue debridement and reconstruction, skeletal stabilization, an external fixator preferably within 6 h of injury prophylactic, and bone grafting with the ultimate goal of achieving bone union without infection and a fully functional pain-free limb. With the improvement in antibiotic use and surgical techniques, the use of intramedullary nails has evolved from being used for low-energy open Gustilo grade 1 and grade 2 fractures to more severe Gustilo grade 3 injuries, with excellent long-term results (4, 5).

The first use of an intramedullary device was documented in ancient Egypt; nevertheless, the first use of intramedullary nailing was documented in Mexico in 1524, and intramedullary nailing was first described in medical journals in the mid-1800s. In the twentieth century, intramedullary nailing evolved in terms of approach, material, cross-section and shape, and reaming technique. Intramedullary nailing was abandoned in favor of plate and screw osteosynthesis in the 1960s; however, a flurry of innovations in the 1970s, 1980s, and 1990s, including flexible reaming, interlocking, and the use of image intensification and titanium nails, led to the introduction of the second-generation intramedullary nailing (6).

Intramedullary nailing is now the standard treatment for long bone fractures, resulting in low infection rates, tiny scars, excellent fracture stability, and fast patient mobilization. Despite the fact that there are various types of intramedullary nails available around the world, our institution has only used Surgical Implant Generation Network (SIGN) nails (1, 5).

The SIGN Standard Intramedullary Nail (Standard Nail) is one solution for these settings. The SIGN is a humanitarian non-profit corporation in Washington, USA that has the goal of providing improved healthcare and proper orthopedic treatment of fractures at little or no cost to people in need worldwide. The SIGN tibial system is a solid intramedullary nail (IMN) with interlocking capability through a mechanical aiming device that enables the placement of proximal and distal interlocking screws without the need for image guidance. This system is currently used by over 200 hospitals in 48 different countries (7).

When clinical examination findings are unclear or unreliable, radiographic union is frequently utilized as a study endpoint and can be an invaluable index. The radiographic union score for tibial fractures (RUST) was developed by Whelan et al. (8) to assess the healing of tibial fractures following intramedullary nailing. The RUST has significantly improved dependability over previously published scores and offers results that are replicable across a wide range of orthopedic specialties and experience levels. There is currently no “gold standard” against which RUST can be measured and this scoring system is a reliable and repeatable outcome measure for assessing tibial fracture healing (9).

### Statement of the problem

Tibial shaft fractures often result in poor outcomes, with delayed union or non-union occurring in up to one-third of cases (10). Even patients with normal union experience residual physical disability, pain, and difficulty returning to work at 1 year post-injury (10, 11). Long-term follow-up studies have shown that 26% of patients report ongoing knee pain, 10% report ankle pain, and 17% report both, correlating with poorer functional outcomes (11). Quality of life remains diminished at 4 and 12 months post-injury compared to pre-injury status, with 44% of patients not regaining full function of the injured leg at 1 year (12). Factors contributing to poor outcomes include high-energy trauma, open fractures, smoking, and comorbidities (10). The poor soft tissue envelope surrounding the tibia, combined with the frequency of high-energy fractures, contributes to healing problems and failed fixation (13).

Intramedullary interlocking nailing is widely considered the preferred treatment for tibial shaft fractures, offering advantages such as early stabilization, mobilization, and high union rates (14). Multiple studies have reported favorable outcomes, with excellent to good results in 90%–96% of cases (14–16). The technique preserves periosteal blood supply; maintains length, rotation, and alignment; and lowers infection and malunion rates (14). Complications such as delayed union, malunion, and non-union are relatively low, occurring in 2%–16% of cases (14, 15). Early weight-bearing and rehabilitation contribute to fracture union (15). However, open injuries with severe soft tissue damage can lead to post-operative infections and non-union (15). Overall, intramedullary interlocking nailing is considered an effective treatment for tibial shaft fractures, allowing earlier fracture union with lower morbidity (16).

Tibia shaft fractures are among the most common emergency orthopedic cases and tend to affect men of economically productive age involved in high-energy trauma (17). Management of tibial fractures in adults is a challenge to orthopedic surgeons due to poor soft tissue coverage and subtle blood supply (18). The aim of tibial shaft fractures treatment is to achieve union with correct anatomic alignment and to gain normal knee and ankle biomechanics as well as regaining early independent ambulation (3). Tibial non-union not only has a negative impact on the patient's quality of life, comparable to advanced hip arthrosis and worse than congestive heart failure, but it also has a significant financial impact, costing twice as much as fractures that healed properly. Despite the various alternatives available, treating tibial non-unions remains a difficult task for surgeons (19).

## Methods and materials

### Study setting and design

Addis Ababa Burn, Emergency, and Trauma (AaBET) Hospital is a major trauma center in Ethiopia. It was established in 2015 as part of St. Paul’s Millennium Medical College. AaBET Hospital has approximately 20,000 to 30,000 emergency visits/year to the hospital and provides emergency and outpatient services and elective and emergency surgeries in the respective departments. It provides fracture care including complex acetabular and pelvic injuries. A health facility-based retrospective cross-sectional study design was employed from February to April 2022.

The study participants were identified using the hospital logbook and the SIGN database. The study reviewed the medical charts and x-rays of patients who had tibial fractures treated with intramedullary nails from 1 March 2015 to October 2020.

The patient's radiological outcomes were assessed by RUST criteria based on anteroposterior and lateral x-rays of the leg at the 12-month follow-up.

### Source population and study populations

All patients with tibial fractures treated with intramedullary nails at AaBET Hospital in the Orthopedic and Traumatology departments were taken as the source population. The study population was all sampled patients with tibial shaft fractures treated with intramedullary nails at AaBET Hospital who fulfilled the inclusion criteria.

### Sample size determination and sampling procedures

A single population proportion sample size determination formula was used based on the confidence interval (CI) approach. A study conducted at Tikur Anbessa Hospital showed that the incidence of tibial shaft fracture was 14%. Thus, p was taken as 14%, with a 95% confidence interval (Z*_α_*_/2_ = 1.96) and a 5% margin of error (d = 0.05). There were 650 patients who underwent operations for tibial fractures in the study period [as counted from the operating room (OR) registration logbook and SIGN database]. Because the sampling was from a finite population size, i.e., *N* = 650, the final sample size calculated using the correctional formula was 144. The final sample size was therefore 160 after 10% for non-respondents was added. The patients in the source population were identified from the hospital log book and SIGN database. The medical charts were arranged in order of their medical record number. A random sampling technique using a generated table of random numbers was used to select the patients for the medical record and x-ray review until the required sample size was obtained. All extra-articular closed and open, Gustilo–Anderson (GA) I, II, and IIIA tibial fractures with/without fibula fracture treated with intramedullary nails with at least 1 year of follow-up were included.

### Data collection and quality assurance

Data were collected from the online SIGN database and patients' medical records using a questionnaire for all the study variables. The questionnaire was developed and adopted from other similar research in a way that will address the objectives of this study. The questionnaire included all the study variables. Prior to data collection, pretesting was done on 5% of the study population. After the pretest, the questionnaire was found to be reliable and sensitive.

The data collected from the SIGN database and patient records were entered into a data abstraction form by four 3rd-year orthopedics residents who understood the objective of the study and the data abstraction variables.

In addition, regular supervision was conducted during data collection to ensure the accuracy and completeness of the data throughout the data collection. The completeness, accuracy, and clarity of each patient's data were checked by the principal investigator before the execution of any data entry process.

Data cleanup for outliers was conducted during and after data entry to control for possible errors.

### Data analysis

Data were collected from the online SIGN surgical database and patient records and then each patient's data were verified, validated, and recorded (cleaned and checked for quality) in a follow-up before analysis. The collected data were entered into the Statistical Package for the Social Sciences (SPSS) version 26 software package for data analysis. Descriptive statistics such as mean, percentage, and standard deviation were determined with corresponding confidence intervals. Frequency and cross-tabulation were used to summarize the descriptive statistics. Means and percentages were used for nominal variables. The degree of association between the dependent and independent variables was determined using binary logistic regression with a CI of 95% and *p*-value of <0.05. Radiological outcome was measured by RUST.

### Operational definitions

Tibial shaft fracture: fractures excluding those within 5 cm of the ankle and knee joint.

Open fracture: when there is direct communication between the fracture hematoma and the external environment, which is sub-classified by Gustilo and Anderson (details are presented in Table 1).

**Table 1:** Overview of Gustilo–Anderson classification for open fractures.

Gustilo–Anderson classification	Energy	Wound size (cm)	Soft tissue	Contamination	Fracture pattern	Periosteal stripping
I	Low energy	<1	Minimal	Clean	Simple fracture pattern with minimal comminution	No
II	Moderate	>1	Moderate	Moderate contamination	Moderate comminution	No
IIIA	High	>10	Extensive	Extensive	Severe comminution or segmental fractures	Yes
IIIB	High	>10	Extensive	Extensive	Severe comminution or segmental fractures	Yes
IIIC	High	>10	Extensive	Extensive	Severe comminution or segmental fractures	Yes

Radiographic union: a RUST of 8 and above.

Non-union: no radiographic union or RUST below 8 12 months after surgery.

RUST: The RUST is a new radiographic fracture assessment instrument that was created to help standardize tibial fracture unions that were treated with intramedullary nails. Cortical bridging and fracture lines are assessed with this score. It has a minimum score of 4 (non-union) and a maximum of 12. A score of 12 indicates complete union. A RUST of 8 indicates union. Scores below this are equated to poor outcomes in this study (details are presented in Table 2).

**Table 2:** Overview of RUST.

Score per cortex	Callus	Fracture line
1	Absent	Visible
2	Present	Visible
3	Present	Invisible

## Result

### Socio-demographic characteristics of the study population

This study included 160 patients with unilateral tibial shaft fractures. The participants comprised 122 (76.3%) men and 38 (23.8%) women. The mean age of participants was 36.38 with a standard deviation of 13.9 (details are presented in Table 3).

**Table 3:** Socio-demographic characteristics of the study participants, AaBET Hospital, 2022.

Variable		Frequency	Percentage
Sex	Male	122	76.3
	Female	38	23.8

### Variables related to past medical history

**Figure 1:**
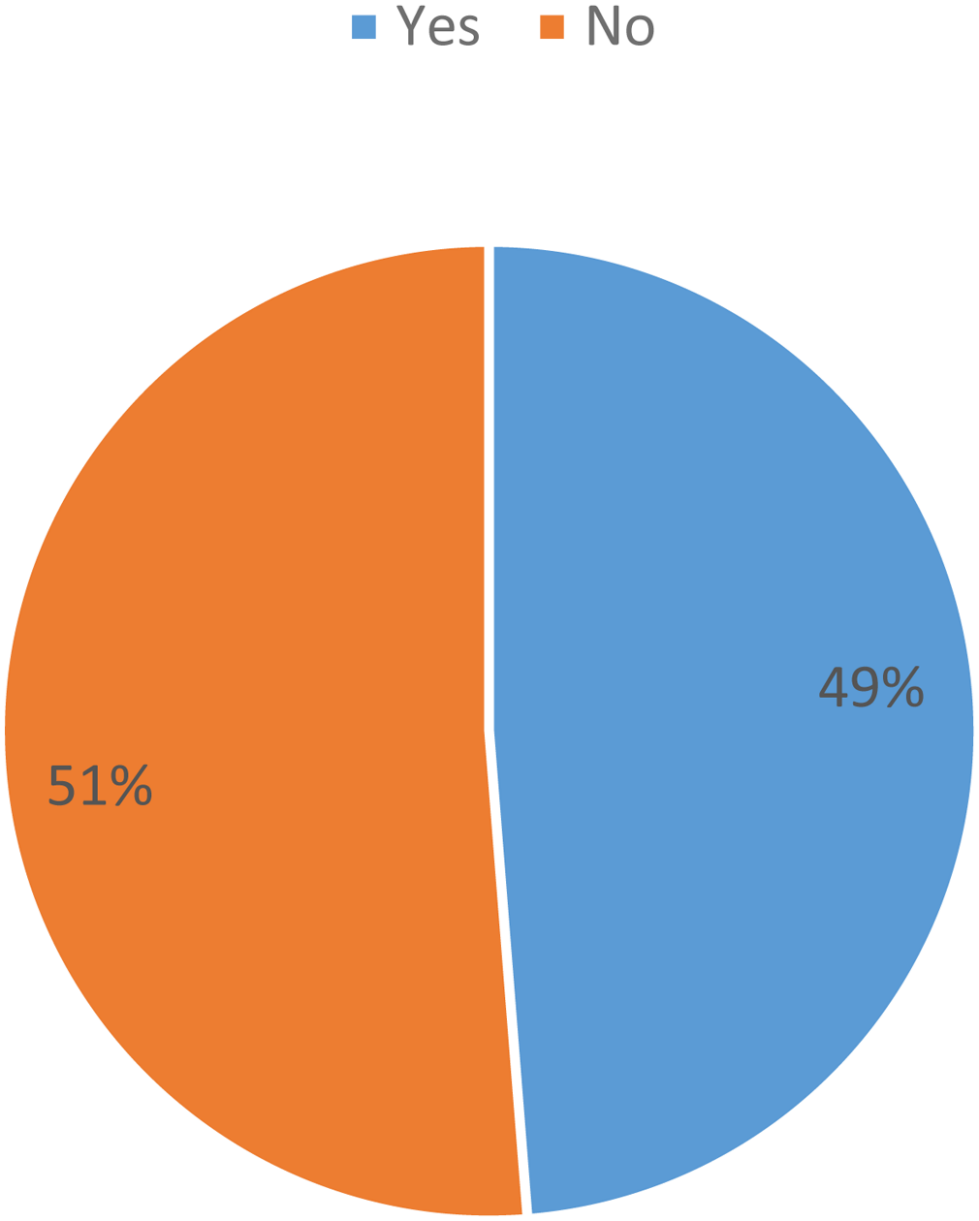
Distribution of participants associated with medical comorbidity among tibial shaft fracture patients treated with intramedullary nail fixation at the AaBET Hospital, Ethiopia, 2022.

**Figure 2:**
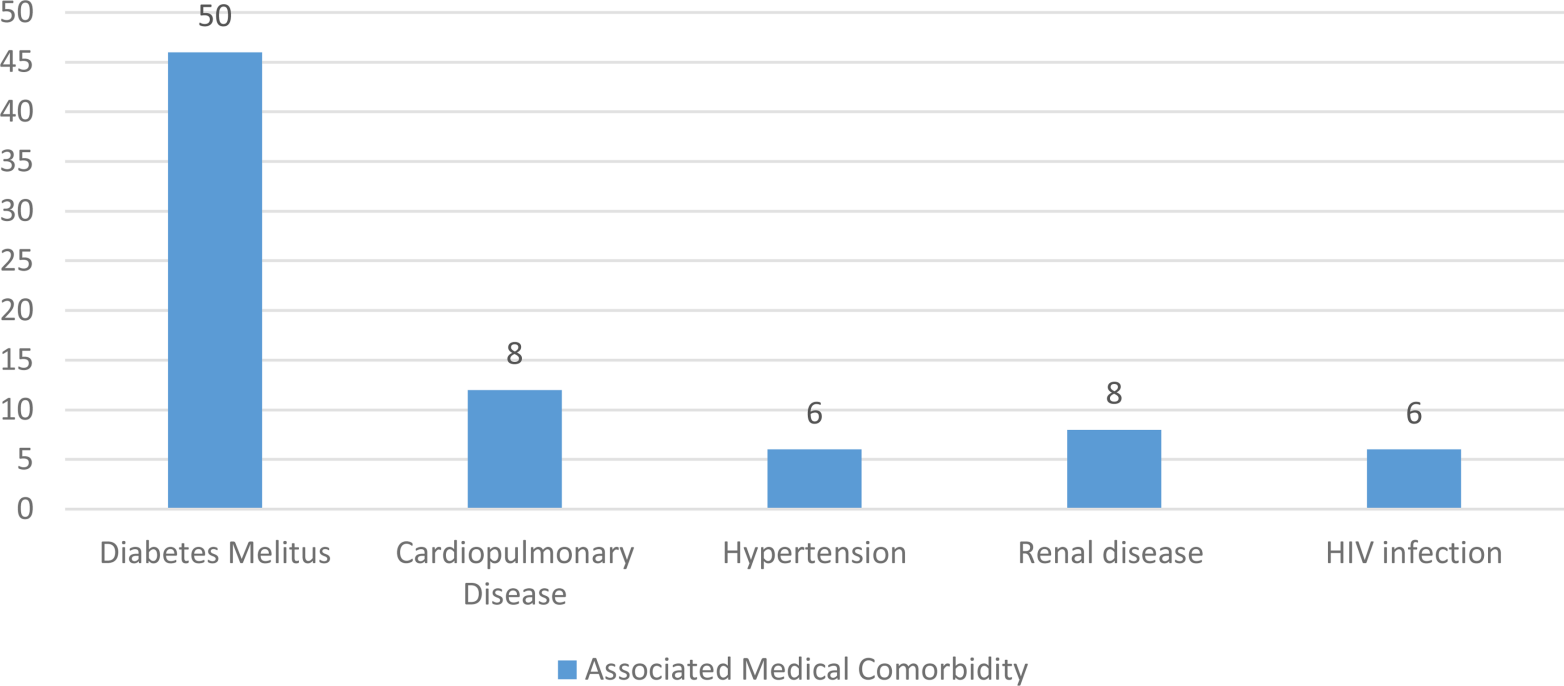
Distribution of patients with a particular type of associated medical comorbidity among tibial shaft fracture patients treated with intramedullary nail fixation at the AaBET Hospital Ethiopia, 2022.

Of the 160 sampled patients included in this study, 78 (48.8%) had a previously diagnosed comorbidity at the time of the tibial shaft fracture. Of the patients with associated comorbidity, 50 (31.3%) had diabetes mellitus (DM) and 8 (5%) had cardiopulmonary diseases, as shown in Figures 1, 2.

### The main causes of tibial shaft fracture and related characteristics

Among the study participants, more than half sustained their injury from a road traffic accident while only eight patients sustained injury due to firearms. The majority of the patients sustained injury to their right extremity. Open fractures accounted for 77 (48.1%) and of these, 29 (18.1%) had a Gustilo–Anderson type I fracture while 32 (20.0%) had a Gustilo–Anderson type III fracture (details are presented in Table 4).

**Table 4:** Major cause of tibial shaft fractures and related characteristics at AaBET Hospital, 2022.

Variable		Frequency	Percentage
Cause of injury	Road traffic accident	101	63.1
	Fall down accident	26	16.3
	Fighting injury	25	15.6
	Firearms	8	5.0
Side of the injured limb	Right	104	65
	Left	56	35
Soft tissue status	Open	77	48.1
	Closed	83	51.9
Type of open fracture	Gustilo–Anderson type I	29	18.1
	Gustilo–Anderson type II	16	10.0
	Gustilo–Anderson type III	32	20.0
Associated injury	Yes	52	32.5
	No	108	67.5
Associated fibular fracture	Yes	119	74.4
	No	41	25.6

**Figure 3:**
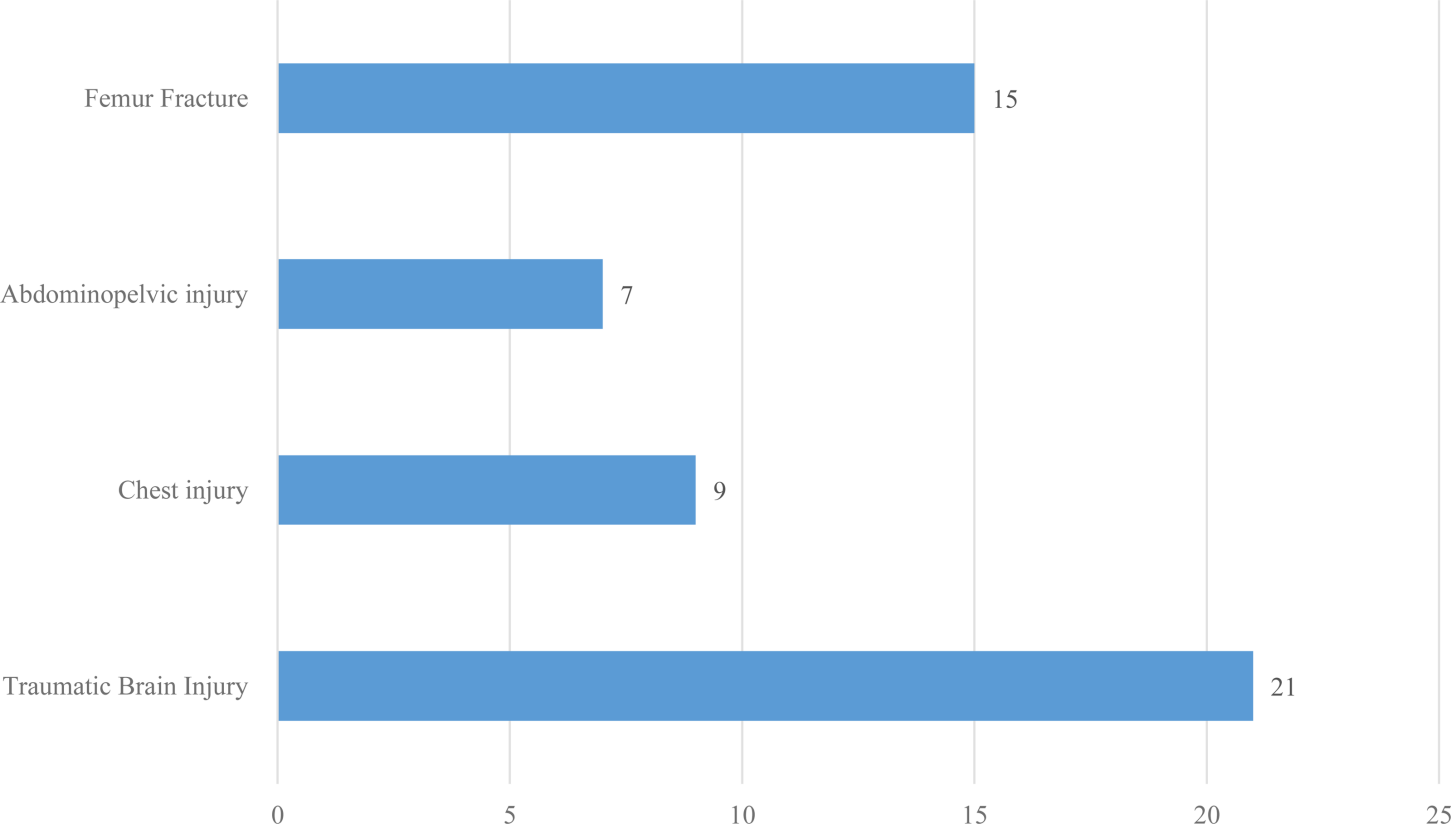
Distribution of patients according to associated injury types among tibial shaft fracture patients treated with intramedullary nail fixation at the AaBET Hospital, Ethiopia, 2022.

One-third of the sampled patients had other associated injuries at the time of presentation with traumatic brain injury being the most common associated injury. Approximately three-quarters of the patients had associated fibular fractures along with tibial fractures (details are presented in Figure 3).

### Intra- and post-operative factors

Among the sampled patients with open fractures, antibiotics were initiated within 3 h in 14 (8.8%) patients and after 24 h in 24 (15%) patients. Antibiotics were continued after definitive fixation for less than 3 days in 33 (20.6%) patients and for more than 7 days in 81 (50.6%) patients. Among the patients with an open fracture, 44 (27.5%) were taken to the operating room for irrigation in debridement in less than 24 h while only 5 (3.1%) were taken to the operating room after 72 h. The closed reduction technique during intramedullary nailing was used in only 26 (16.3%) cases while the open reduction technique was used in the remaining patients (details are presented in Table 5).

**Table 5:** Factors related to intra- and post-operative factors, AaBET Hospital, 2022.

Variable		Frequency	Percentage
Time from trauma to initiating antibiotics	No antibiotics initiated	83	51.8
	Within 3 h	14	8.7
	4–24 h	39	24.3
	After 24 h	24	15
Post-operative antibiotics (days)	<3	33	20.6
	4–7	46	28.7
	>7	81	50.6
External fixation before IM nail	Yes	12	7.5
	No	148	92.5
Reduction technique	Open	134	83.8
	Closed	26	16.3

**Figure 4:**
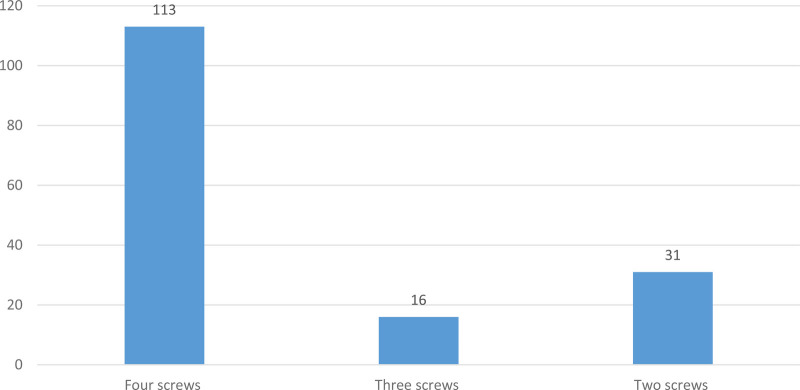
Distribution of patients according to the total number of interlocking screws used among tibial shaft fracture patients treated with intramedullary nail fixation at the AaBET Hospital, Ethiopia, 2022.

Four interlocking screws were used during intramedullary nailing in 113 (70.6%) patients while two screws were used in 31 (19.4%) patients, as shown in Figure 4.

### Complications associated with intramedullary nail fixation in tibial shaft fracture patients

Post-operative infection after intramedullary nailing was found in 47 (29.3%) patients. Of these patients with a post-operative infection, 32 (20.0%) had a deep infection. Implant failure was recorded in 19 (11.9%) patients and of these, screw breakage was the common type of implant failure (details are presented in Table 6).

**Table 6:** Post-operative complications of tibial shaft fracture patients treated with intramedullary nail fixation, AaBET Hospital, 2022.

Variable		Frequency	Percentage
Post-operative infection	Yes	47	29.3
	No	113	70.7
Type of post-operative infection	No infection	113	70
	Superficial infection	12	7.5
	Deep infection	32	20.0
	Contaminated (COM)	3	1.9
Implant failure	Yes	19	11.9
	No	141	88.1
Type of implant failure	No implant failure	141	88.1
	Screw breakage	12	7.5
	Nail breakage	7	4.4

### Multivariable analysis of factors associated with radiological outcome

Using the independent variables that were significant in the bivariate analysis, namely, the presence of DM as comorbidity, Gustilo–Anderson type III open fracture, two interlocking screws, and post-operative antibiotics for 4–7 days and for more than 7 days, post-operative infection, and deep infection, multivariable logistic regression was carried out by including all the variables to adjust for confounders.

As depicted in Table 7, in the stepwise multiple logistic regression, it was found that patients with DM as an associated medical comorbidity were 3.8 times more likely to have non-union [adjusted odd ratio (AOR) (95% CI): 3.85 (1.07–14.08)]. Patients with GA type III open fractures were 17.4 times more likely to have non-union [AOR (95% CI): 17.4 (3.11–97.72)]. This study found that patients who developed post-operative infections were 6.1 times more likely to have non-union [AOR (95% CI): 6.15 (1.44–26.3)].

**Table 7:** Multivariable analysis of factors associated with a poor radiological outcome, AaBET Hospital, 2022.

Variable	Radiological outcome		COR (95% CI)	AOR (95% CI)	*P*-value
	Union	Non-union			
Gustilo–Anderson III					
Yes	19	13	2.96 (1.28–6.82)	17.4 (3.11–97.7)	0.003
No	104	24	1.00	1.00	
Diabetes mellitus					
Yes	19	29	14.77 (6.16–35.45)	3.88 (1.07–14.08)	0.030
No	104	8	1.00		
Post-op infection					
Yes	20	27	13.90 (5.8–33.16)	6.15 (1.44–26.3)	0.014
No	103	10	1.00		

COR, crud odd ratio; AOR, adjusted odd ratio.

However, the remaining independent variables that were found to be statistically significant in the bivariate analysis were not significantly associated with non-union in the stepwise multivariate logistic regression (details are presented in Table 7).

## Discussion

This study investigated the prevalence of poor radiological outcomes and associated factors among patients with tibial shaft fractures treated with IMNs.

Men made up 76.3% of the participants in this study, and the average age at tibial shaft fracture was 36.3. Road traffic accidents were responsible for 63.1% of the cases. These findings are in line with those of a research study conducted at Black Lion Hospital (20), in which the male-to-female ratio was 3:1, the average age at fracture was 35 years, and road traffic accidents accounted for 48% of the cases. A Danish study (21) found that the average age at the time of tibial shaft fracture was 38.5 years, with men (63.8%) being the most affected. Similarly, Larsen et al. (21) discovered that 74% of cases involved traffic accidents. This indicates that tibial shaft fractures are more common in men of working age who are involved in high-energy trauma.

In this study, open fractures accounted for 77 (48.1%) cases, and nearly a third of the patients had a fibular fracture in addition to tibial fractures. This figure is comparable to a previous study (22) in which 53% of the tibial fractures were open, while 95% of the fibular fractures were discovered.

In this study, participants had a 23.1% chance of having a poor radiological outcome. This was similar to a previous study that found that the rate of non-union after tibial shaft fractures treated with IMNs ranged from 5% to 33% (23). This means that a poor radiological outcome after tibial shaft fractures treated with IMN is a major concern as they largely afflict young productive people and surgeons have difficulty treating tibial fracture non-union.

The current study also found that Gustilo–Anderson type III open fractures and post-operative infections are associated with poor radiological outcomes. However, these findings are in contrast to other similar studies. A study was previously conducted to evaluate the results of intramedullary nailing with and without reaming for open tibial shaft fractures (23). In this study, a total of 94 open tibial shaft fractures were divided into two groups with 50 (9 GA I, 18 GA II, 16 GA IIIA, and 7 GA IIIB fractures) patients treated with reamed intramedullary nails and 44 patients without reaming and the RUST was used to assess their radiological outcomes. All of the procedures were conducted by or were under the direct supervision of the senior surgeons. They found that four (one type I, one type II, and two type IIIB) (9%) patients treated with reaming had non-union and there were two cases of post-operation infections. However, none of these had an association with poor radiological outcomes. Similarly, a study conducted in South Africa (23) retrospectively evaluated the clinical data and radiographs of 74 Gustilo–Anderson type I–IIIA open tibial shaft fractures and found no difference in the rate of poor radiological outcomes among the types of open fractures. These findings are in line with a study that found significant differences between group I and group II in erythrocyte sedimentation rate, C-reactive protein, mean platelet volume (MPV) blood test values, and irisin, indicating a relationship between fracture healing and mean platelet volume (24). The fracture fixations were performed by a senior surgeon and negative pressure wound therapy was applied for GA III fractures. In contrast to these studies, all of the cases in our study were treated with reamed intramedullary nails, most of the fixations were done by residents, and there was no application of negative pressure wound therapy for GA III tibial shaft fractures. This could indicate that senior surgeon involvement and negative wound pressure may be crucial in cases of GA III tibial shaft fractures and in preventing post-operative infections.

A cohort study in Edinburgh conducted on 647 patients investigated risk variables for aseptic non-union after tibial diaphyseal fractures treated with intramedullary nailing (25). There were 41 non-unions (6.3%), indicating that non-steroidal anti-inflammatory drug (NSAID) use, a high-energy injury mechanism, open fractures, and superficial infection were all independently linked to a higher risk of non-union in patients undergoing IM nailing for a tibial diaphyseal fracture. However, the individuals in this study had a 23.1% likelihood of having a bad radiological outcome, despite the fact that the overall figure is higher. Our investigation found that post-operative infection is a risk factor that increases the odds of poor treatment outcomes, which strongly supported a previous study conducted on human bone tissue in the bone healing process that found intense staining in compact bone tissue and muscle tissue and hypertrophic vascular endothelium occurred within the Haversian canal (26). Furthermore, another study (27), which examined 27 diabetic patients who had a tibial fracture treated with a reamed intramedullary nail compared to a non-diabetic control group, found no difference in the rate of complications between the diabetic and non-diabetic patients. In contrast with the findings of this study, our study revealed that diabetes patients are 3.8 times more likely to have poor radiological outcomes. This disparity could be attributable to the small sample size employed in these studies.

### Strengths and limitations of the study

Facility-based cross-sectional study designs offer valuable insights and are practical for assessing health-related issues within specific populations, such as radiological outcomes following intramedullary nailing of tibial shaft fractures, but also have limitations regarding causality and bias. Researchers must consider these issues when interpreting the findings as the study's population was from a public institution and the sample size was small, making it difficult to establish cause-and-effect relationships.

## Conclusion

In conclusion, poor radiological outcomes following intramedullary nailing of tibial shaft fractures are a major concern. This study determined that the prevalence of poor radiological outcomes among tibial shaft fracture patients treated with intramedullary nail fixation was moderate and identified associated factors that increased the odds of poor radiological outcomes, namely, Gustilo–Anderson type III open fractures, diabetes mellitus, and post-operative infections. Therefore, surgeons should improve their assessment and evaluation of patients with infection signs and use negative wound pressure for GA III fractures. Policymakers should enhance road safety measures to reduce traffic accidents, which accounted for half of tibial shaft fracture cases in the study setting. Healthcare providers should emphasize close follow-ups with patients with diabetes mellitus to reduce poor radiological outcomes. This will help reduce the incidence of injuries and improve overall health outcomes.

Similar studies should be carried out on a nationwide scale to identify and address the factors associated with poor radiological outcomes in tibial shaft fractures.

## Data Availability

The raw data supporting the conclusions of this article will be made available by the authors, without undue reservation.
